# Antiracism and positive intergenerational (infant) outcomes: A county-level examination of low birth weight and infant mortality

**DOI:** 10.1073/pnas.2320299121

**Published:** 2024-04-01

**Authors:** Tiffany N. Brannon

**Affiliations:** ^a^Department of Psychology, University of California, Los Angeles, CA 90095-1563

**Keywords:** antiracism, low birth weight, infant mortality, mental/physical health, intergroup

## Abstract

Racism is associated with negative intergenerational (infant) outcomes. That is, racism, both perceived and structural, is linked to critical, immediate, and long-term health factors such as low birth weight and infant mortality. Antiracism—resistance to racism such as support for the Black Lives Matter (BLM) movement—has been linked to positive emotional, subjective, and mental health outcomes among adults and adolescents. To theoretically build on and integrate such past findings, the present research asked whether such advantageous health correlations might extend intergenerationally to infant outcomes? It examined a theoretical/correlational process model in which mental and physical health indicators might be indirectly related to associations between antiracism and infant health outcomes. Analyses assessed county-level data that measured BLM support (indexed as volume of BLM marches) and infant outcomes from 2014 to 2020. As predicted, in the tested model, BLM support was negatively correlated with 1) low birth weight (*N_counties_* = 1,445) and 2) mortalities (*N_counties_* = 409) among African American infants. Given salient, intergroup, policy debates tied to antiracism, the present research also examined associations among White Americans. In the tested model, BLM marches were not meaningfully related to rates of low birth weight among White American infants (*N_counties_* = 2,930). However, BLM support was negatively related to mortalities among White American infants (*N_counties_* = 862). Analyses controlled for structural indicators of income inequality, implicit/explicit bias, voting behavior, prior low birth weight/infant mortality rates, and demographic characteristics. Theory/applied implications of antiracism being linked to nonnegative and positive infant health associations tied to both marginalized and dominant social groups are discussed.

The people marched, and I had never known that there could be rivers such as this, and as protesters chanted and stomped, as they grimaced and shouted and groaned, tears burned my eyes…The revelation that Black Americans were not alone in this, that others around the world believed that Black Lives Matter broke something in me, some immutable belief I’d carried with me my whole life. This belief beat like another heart—*thump*—in my chest from the moment I took my first breath as an underweight, two-pound infant after my mother, ravaged by stress, delivered me at 24 wk. It beat from the moment the doctor told my Black mother her Black baby would die. *Thump.*
Jesmyn Ward (1 September 2020)

Award-winning novelist, Jesmyn Ward’s words in the above excerpt capture the impact of witnessing people march in support of the Black Lives Matter (BLM) movement. In particular, her prose suggests that witnessing such support for antiracism can provide a poignant contrast to experiences linked to systemic racism. Ward’s words represent racism as a complex, living entity, assigning it a heartbeat (e.g., “thump”) that has beat since she was a low birth weight infant born to a “Black mother”. Aligned with Ward’s recognition of the longstanding impact of racism on her life, which she traces back to her infancy, past research has shown that racism is associated with negative health outcomes among infants (i.e., low birth weight, mortality; 1, 2). More recent research has examined associations between antiracism such as support for and/or exposure to support for the BLM movement and emotional, mental, and subjective health outcomes among adults and adolescents (3, 4). Building on such research that suggests antiracism can benefit health and well-being, the present research examined associations between exposure to BLM support (indexed as county-level BLM marches) and two consequential health outcomes among infants—low birth weight and mortality. Specifically, the present research theorizes that like racism, antiracism is likely linked to complex and interrelated individual, social, and structural factors (3, 4). Consistent with past research, it theorizes that antiracism is linked to such factors in ways that can be positively associated with well-being (see refs. 3 and 4). In addition, extending such past research, it predicts that such factors may be related to associations between antiracism and health indicators that go beyond individual-level measures (e.g., county-level outcomes).

Across county-level analyses, it was predicted that antiracism would be associated with smaller percentages of low birth weight infants born to non-Hispanic, African American mothers. It was also predicted that antiracism would be related to a lower rate of mortalities among African American infants. Importantly, past research has linked racism to adverse effects on mental and physical health, while more recent research has revealed associations between antiracism and advantageous effects on mental, emotional, and physical health (3–6). To theoretically build on and integrate such past findings the present research examined theoretical/correlational process (mediation) analyses in which county-level mental and physical health indicators might be indirectly related to associations between antiracism and the noted infant health outcomes. Given salient policy debates tied to intergroup effects of antiracism on socially dominant groups the present research also examined the noted analyses among White Americans. The present research did not expect antiracism to be associated with negative consequences for infant outcomes among White Americans. This expectation is consistent with research which has found that a) racism can negatively impact socially marginalized groups (e.g., African Americans) as well as dominant groups (e.g., White Americans; 7, 8) and b) antiracism can be associated with positive and/or neutral consequences across social groups (3). All analyses a) used BLM protest data and rates of low birth weight and mortalities among infants from 2014 to 2020, and b) controlled for structural indicators of income inequality, implicit/explicit bias, voting behavior, prior low birth weight/infant mortality rates, and demographic characteristics.

## Results

To test the primary predictions theoretical/correlational process (mediation), analyses were run [PROCESS (version 4.0) macro, SPSS; 9]. The predictions were tested separately for African Americans and White Americans infant outcomes. All models included BLM support as the predictor variable, composite measures of mental/physical health indicators as the mediator, and the noted covariates.

### Infant Outcomes.

#### (African Americans).

**Fig. 1.**
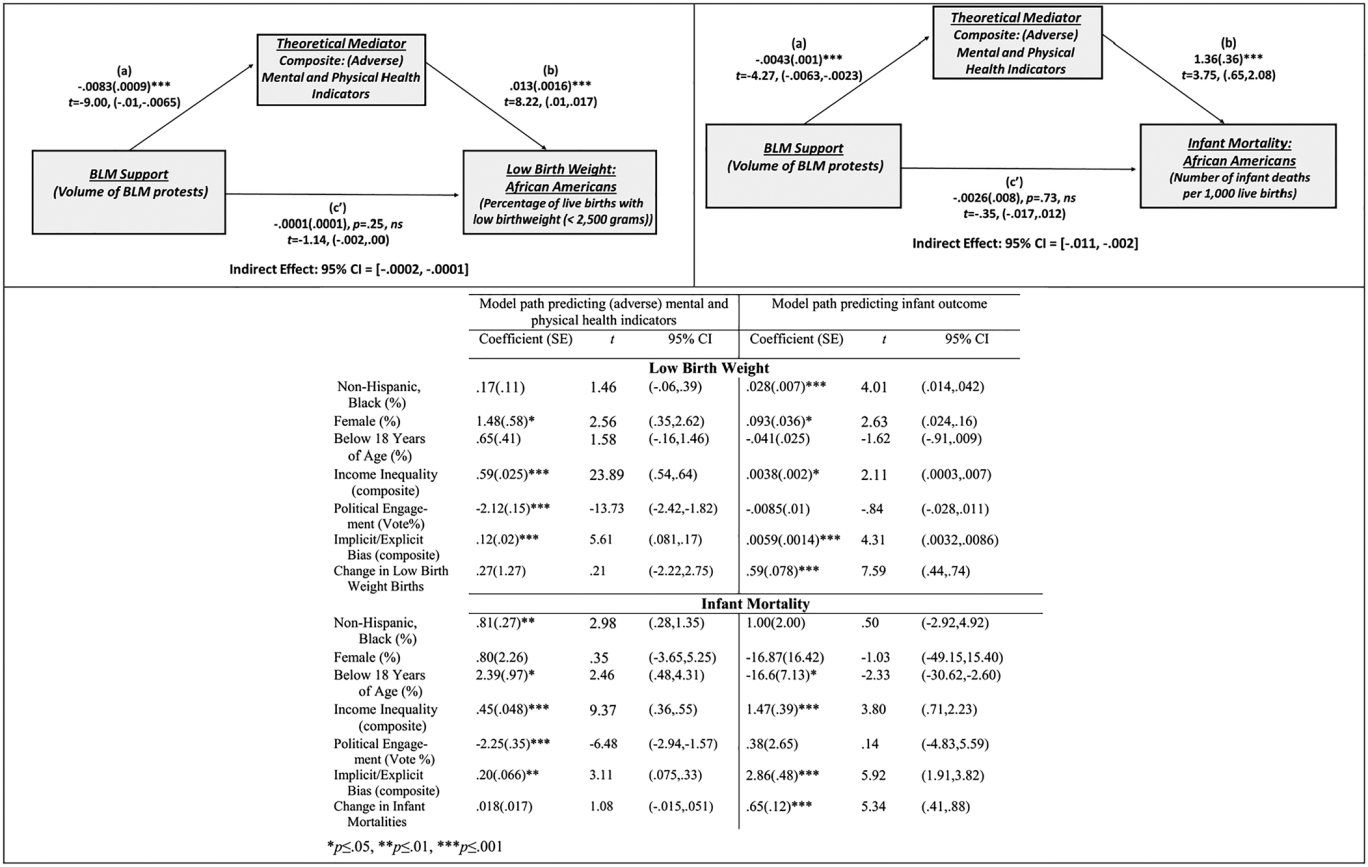
Process Model: (County-Level Analysis—African Americans) Showing the indirect effect of BLM support on infant outcomes through mental/physical health indicators.

As predicted, and shown in Fig. 1, a significant indirect effect of BLM support on the infant outcomes through the composite measure of mental/physical health indicators was observed: low birth weight, b = −0.0001, SE = 0.00, CI = −0.0002, −0.0001 and infant deaths, b = −0.0059, SE = 0.0022, CI = −0.011, −0.002.

#### (White Americans).

**Fig. 2.**
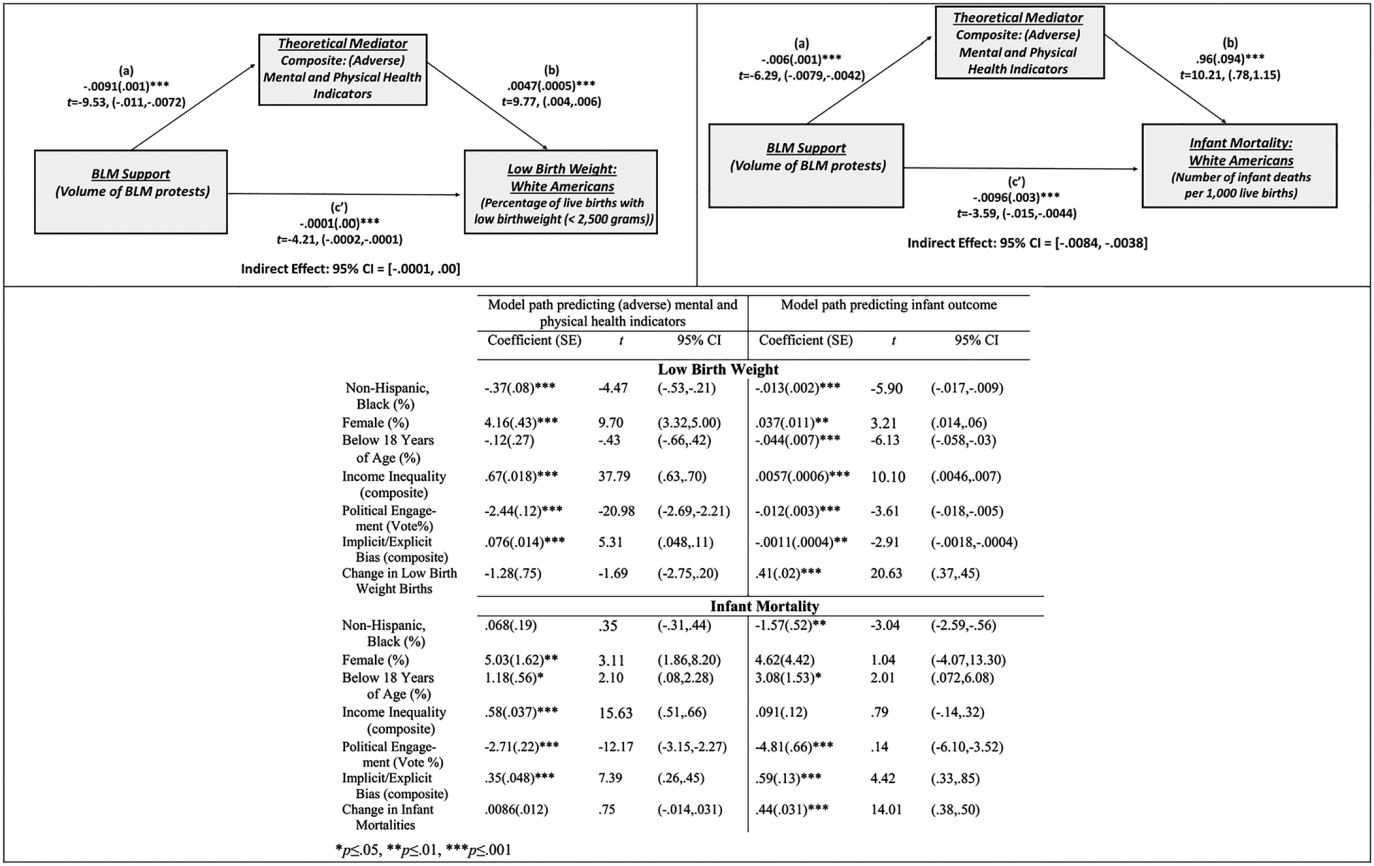
Process Model: (County-Level Analysis—White Americans) Showing the indirect effect of BLM support on infant outcomes through mental/physical health indicators.

As shown in Fig. 2, the analysis revealed a not meaningful indirect effect of BLM support on low birth weight through the composite measure of mental/physical health indicators, b = −0.00, SE = 0.00, CI = −0.0001, 0.00. However, the analysis revealed a significant indirect effect of BLM support on infant deaths through the composite measure of mental/physical health indicators, b = −0.0058, SE = 0.0012, CI = −0.0084, −0.0038.

## Discussion

The present research provides some evidence that antiracism, indexed as county-level BLM marches, can be associated with intergenerational, infant outcomes. The present research has limitations related to the cross-sectional and correlational nature of the data. However, the present research has strengths tied to examining: two distinct yet related infant outcomes—low birth weight and mortalities, effects among marginalized and dominant social groups, associations of antiracism while controlling for multiple factors including implicit/explicit bias and structural income inequality. Given salient policy debates tied to antiracism in state laws, school curriculum, and organizational trainings the present research suggests that efforts to signal support for groups that are marginalized like African Americans might be associated with positive county or community-level health outcomes. It also suggests that antiracism efforts are not necessarily related to zero-sum intergroup consequences. Aligned with the opening quote by Ward, the results suggest that antiracism efforts such as BLM marches might serve as a potentially powerful and complex affirmation of the humanity and dignity of social groups that have long been the target of racism and dehumanization.

## Materials and Methods

All measures were tested at the county level and are derived from publicly available data sources.

### BLM Support (Volume of Protest).

Data on BLM protests were obtained from an open-source dataset (10, see ref. 11). To match the data on infant health outcomes that are aggregated from 2014 to 2020, data on BLM protests that occurred between August 2014 (which is the earliest timepoint available in the dataset) and December 2020 were included for analyses.

### Composite: (Adverse) Mental and Physical Health Indicators.

Measures of mental and physical health were assessed in 2020 and obtained using 2023 County Health Rankings National Data (CHRND; 12). The mental health measures included poor mental health days, insufficient sleep, and mental distress. The physical health measures included poor/fair health, poor physical health days, adult smoking, adult obesity, diabetes prevalence, frequent physical distress, and excessive drinking. The standardized composite of these measures averaged .0006 (SD = 0.76).

### Infant Outcomes.

The 2023 CHRND was also used to obtain data on the percentage of live births with low birth weight (<2,500 g) and infant deaths per 1,000 live births, both aggregated from 2014 to 2020. The average percentage of low birth weight infants for African Americans was 0.14 (SD = 0.031) and 0.074 (SD = 0.015) for White Americans. The average number of infant mortalities for African Americans was 12.06 (SD = 3.63) and 5.42 (SD = 1.79) for White Americans.

### Covariates.

Covariates were obtained from the 2017 and 2023 CHRND and Project Implicit Demo Website Datasets (13). Demographics included percentage of the population: below 18 y of age (range: 0.05 to 0.38, M = 0.22, SD = 0.033), self-identifying as non-Hispanic Black/African American (range: 0.001 to 0.82, M = 0.091, SD = 0.14), female (range: 0.26 to 0.57, M = 0.50, SD = 0.021). A standardized composite for income inequality included unemployment, income inequality, children in poverty, percent of population without some college, and children in single-parent households; M = −0.013, SD = 0.70. Voter turnout gauged political engagement, M = 0.65, SD = 0.096. Change in infant birth weight (M = 0.0009, SD = 0.012) and mortalities (M = −0.36, SD = 1.42) captured difference scores between these variables in the 2017 and 2023 CHRND. A standardized implicit/explicit bias composite captured anti-Black bias, M = 0.01, SD = 0.74.

## Supplementary Material

Appendix 01 (PDF)

## Data Availability

Previously published data were used for this work [County Health Rankings National Data, https://www.countyhealthrankings.org/explore-health-rankings/rankings-data-documentation] (10).

## References

[1] K. L. Karvonen , Structural racism is associated with adverse postnatal outcomes among Black preterm infants. Pediatric Res. **94**, 371–377 (2023). 10.1038/s41390-022-02445-6 PMC9795138 36577795

[2] J. W. Collins Jr., R. J. David, Racial disparity in low birth weight and infant mortality. Clin. Perinatol. **36**, 63–73 (2009). 19161865 10.1016/j.clp.2008.09.004

[3] T. N. Brannon, Racism hurts, can antiracism heal?: Positive mental health correlates of antiracist engagement. PNAS Nexus **2**, 1–10 (2023). 10.1093/pnasnexus/pgad309 PMC10548497 37799326

[4] A. Baskin-Sommers , Adolescent civic engagement: Lessons from Black lives matter. Proc. Natl. Acad. Sci. U.S.A. **118**, 1–3 (2021). 10.1073/pnas.2109860118 PMC8521674 34607958

[5] D. R. Williams, R. Williams-Morris, Racism and mental health: The African American experience. Ethn. Health **5**, 243–268 (2000). 11105267 10.1080/713667453

[6] C. S. Levine, H. R. Markus, M. K. Austin, E. Chen, G. E. Miller, Students of color show health advantages when they attend schools that emphasize the value of diversity. Proc. Natl. Acad. Sci. U.S.A. **116**, 6013–6018 (2019). 30858317 10.1073/pnas.1812068116 PMC6442588

[7] J. C. Eichstaedt , The emotional and mental health impact of the murder of George Floyd on the US population. Proc. Natl. Acad. Sci. U.S.A. **118**, 1–5 (2021). 10.1073/pnas.2109139118 PMC8488615 34544875

[8] W. Berry Mendes, H. M. Gray, R. Mendoza-Denton, B. Major, E. S. Epel, Why egalitarianism might be good for your health: Physiological thriving during stressful intergroup encounters. Psychol. Sci. **18**, 991–998 (2007). 17958714 10.1111/j.1467-9280.2007.02014.x PMC2430625

[9] A. F. Hayes, Introduction to Mediation, Moderation, and Conditional Process Analysis (The Guilford Press, ed. 3, 2022). http://www/guiford.com/p/hayes3. Accessed 5 October 2023.

[10] H. Yan, Z. Dunivin, F. Rojas, J. Ince, Black lives matter protests shift public discourse. Retrieved from osf.io/ubptz (2022). Accessed 18 September 2023. 10.1073/pnas.2117320119 PMC8915973 35239433

[11] Z. O. Dunivin, H. Y. Yan, J. Ince, F. Rojas, Black Lives Matter protests shift public discourse. Proc. Natl. Acad. Sci. U.S.A. **119**, 1–11 (2022). 10.1073/pnas.2117320119 PMC8915973 35239433

[12] University of Wisconsin Population Health Institute, County Health Rankings & Roadmaps National Data, https://www.countyhealthrankings.org/explore-health-rankings/rankings-data-documentation. Accessed 6 June 2023.

[13] K. Xu , Project implicit demo website datasets. 10.17605/OSF.IO/Y9HIQ (6 February 2023). Accessed 11 January 2024.

