# Supplementary material for: Antiracism and positive intergenerational (infant) outcomes: A county-level examination of low birth weight and infant mortality
Source: Proc Natl Acad Sci U S A. 2024 Apr 1;121(15):e2320299121. doi: 10.1073/pnas.2320299121 (PMC11009635; doi:10.1073/pnas.2320299121)
Supplement: Supplementary file 1 — Appendix 01 (PDF) [file pnas.2320299121.sapp.pdf]

PNAS

Supporting Information for

Anti-racism and Positive Intergenerational (Infant) Outcomes: A County-level Examination of Low-birth Weight and Infant Mortality

Tiffany N. Brannon*

Department of Psychology, University of California, Los Angeles

Email: tbrannon@ucla.edu

Author Contributions: Theorizing, data analysis, interpretation and writing by Tiffany N. Brannon

Competing Interest Statement: No interest to disclose

Classification: Psychological and Cognitive Sciences

Keywords: antiracism, low birth weight, infant mortality, mental and physical health

This PDF file includes:

Supporting text for Materials and Methods

Supporting Information Text

Materials and Methods

BLM Support (County-level Protest). The number of protests in counties ranged from 0 to 251. Conceptually, the timeframe from 2014 to 2020 is meaningful in the BLM movement as it encompasses heightened periods of protests (see S1). In particular, past research by Dunivin and colleagues (S1, p.3) has demonstrated the following about the specific timeframe:

> The four largest spikes in collective attention given to antiracism reflect major protest events. The first spike occurs in late 2014, following the hearings for the killers of Michael Brown and Eric Garner, which did not result in indictments, and the killing of Tamir Rice. The second spike occurs in July 2016, which are protests following the killings of Alton Sterling and Philando Castile. The third spike appears in August 2017, corresponding to counterprotests following the Unite the Right rally in Charlottesville. The largest spike is in 2020, which is related to the murder of George Floyd.

Composite: Mental and Physical Health Indicators. CHRND is administered by the Wisconsin Population Health Institute and Robert Wood Johnson Foundation. The mental and physical health measures were standardized and averaged to create a composite, alpha=.92 and .83, respectively. The standardized measures were averaged to create a composite, r=.79. The standardized composite of these measures ranged from -2.24 to 2.89.

Infant Outcomes. No additional weighting was done to the data for analysis. As documented (see S2) the available data for outcomes vary based on county reporting practices including data suppression in low number of cases (e.g., concerns about anonymity). All available data were used in the analyses. The percentage of low birth weight infants ranged from: .044 or 4.4% to .30 or 30% for African Americans and .029 or 2.9% to .22 or 22% for White Americans. The number of infant mortalities ranged from: 4.17 to 37.74 for African Americans and 1.67 to 15.32 for White Americans.

Covariates. For the income inequality variable, a factor analysis confirmed that the variables were associated with one construct (all loadings≥.65), eigenvalue=2.85, which accounted for 56.89% of the variance. The income inequality variables were standardized and then averaged, alpha=.81. The income inequality variables were derived from 2017-2021 data. Voter turnout was assessed as the percentage of the population who voted in the 2016-2020 U.S. Presidential elections. Change in infant birth weight and mortalities captured difference scores between these variables in the 2017 and 2023 CHRND. The 2017 CHRND included data on infant birth weight from 2008-2014 and infant mortalities from 2007-2013. The difference scores capture the change in these variables for births across race/ethnicity as the 2017 CHRND did not provide separate data on infant outcomes by race/ethnicity. The standardized implicit/explicit bias composite (alpha=.72) ranged from -6.18 to 6.35. The composite included implicit association measures (d-scores), explicit White/Black preferences, and White/Black feeling thermometers. The data were aggregated from 2014-2020. Standard exclusion criteria were applied to the implicit association data (S3).

References

S1. Z.O. Dunivin, H.Y. Yan, J. Ince, & F. Rojas, Black Lives Matter protests shift public discourse. Proceedings of the National Academy of Sciences, 119(10), 1-11 (2022).

S2. County Health Rankings National Data, https://www.countyhealthrankings.org/explore-health-rankings/rankings-data-documentation

S3. A.G., Greenwald, B.A. Nosek, & M.R., Banaji, M. R. Understanding and using the Implicit Association Test: I. An improved scoring algorithm. *Journal of Personality and Social Psychology,* 85(2), 197–216. https://doi.org/10.1037/0022-3514.85.2.197 (2023).
